# Working With Patients Living With Dementia, Delirium and Behaviours of Concern: An Exploration into the Impact on Allied Health Staff Wellbeing

**DOI:** 10.1177/14713012251408675

**Published:** 2026-01-12

**Authors:** Taree Gibson, Melissa Roberts, Sarah C. Milne, Abby M. Foster

**Affiliations:** 1 Physiotherapy Department, 2538 Monash Health, Melbourne, VIC, Australia; 2 Allied Health Workforce, Innovation, Strategy, Education & Research (WISER) Unit, 2538 Monash Health, Melbourne, VIC, Australia; 3 Bruce Lefroy Centre, Murdoch Children’s Research Institute, Melbourne, VIC, Australia; 4 School of Primary and Allied Health Care, 2541 Monash University, Melbourne, VIC, Australia; 5 Department of Paediatrics, 2281 University of Melbourne, Melbourne, VIC, Australia; 6 School of Allied Health, Human Services & Sport, 2080 La Trobe University, Melbourne, VIC, Australia

**Keywords:** dementia, delirium, allied health, wellbeing, person—centred care

## Abstract

**Purpose:** People living with delirium, dementia and exhibiting behaviours of concern (DDBoC) are becoming increasingly common in hospitals. Allied Health (AH) are a group of professionals who frequently work with people with DDBoC in the inpatient setting to support their rehabilitation goals. Little is known about the effect working with DDBoC has on the mental health and wellbeing of AH staff. The aim of this study was to examine the impact of working with patients living with DDBoC on AH staff on inpatient wards. **Materials and methods:** This study used a mixed methods concurrent triangulation design with three data collection methods: (i) retrospective audit (Jan 2021 to Dec 2022) of clinical incident data to establish prevalence of incidents related to patients with DDBoC and involving AH; (ii) cross-sectional online survey of AH staff wellbeing, and exposure to and experience with patients living with DDBoC; and (iii) semi-structured one-on-one interviews with AH staff exploring experiences working with DDBoC. The study period was between April and June 2023. **Results:** Thirty-nine incidents related to DDBoC involving AH staff were reported during the study period. Survey participants reported frequent exposure to DDBoC (44% reported >5 encounters in the past month) and high levels of support seeking (n = 35/50; 70%). Twenty-six percent (n = 13/50) of participants reported low levels of resilience and 28% (n = 14/50) reported high levels of burnout. Analysis of interview transcripts yielded four key themes: ‘What we experience’, ‘How we are impacted’, ‘How we manage’ and ‘How we work together’. Despite most participants reporting normal levels of stress (n = 35; 70%), interview data suggested senior staff may experience and respond to stress differently. Work in this area impacted on team communication, confidence, and response to external factors outside team control. **Conclusions:** Working with DDBoC has a complex effect on AH staff wellbeing, eliciting feelings of reward, empathy, stress, fatigue and moral distress where person-centred care cannot be provided. AH staff appear to under-report incidents and require strategies to support their wellbeing when working with this patient population.

## Background

Dementia is an umbrella term used to describe a collection of neurocognitive disorders which affect thinking, memory and progressively impact a person’s activities of daily living (World Health Organization, 2022). In contrast, delirium is an acute change in mental status characterised by fluctuating levels of alertness, attention and cognition (ACSQHC, 2021). Dementia is a key predisposing risk factor in the development of delirium (Han et al., 2022). Whilst not always a feature of presentation, delirium and dementia are frequently associated with changed behaviours; which may manifest in response to unmet need, environment or impaired communication; and should be considered from an individual, person-centred perspective (Cunningham, Healy & Macfarlane, 2022). Behaviours causing distress or introducing risk may be referred to as behaviours of concern (BoC), and can include aberrant verbalisations, agitation, confusion and disorientation, verbal or physical aggression, wandering, intrusiveness, social inappropriateness and/or hyper fixation (Cunningham, Healy & Macfarlane, 2022).

In the inpatient rehabilitation setting, patients with dementia and/or delirium, and exhibiting behaviours of concern (DDBoC), are managed by the multidisciplinary team who coordinate care towards rehabilitation goals established with the patient and their family/carers (Department of Health, 2023; Gupta et al., 2021). The multidisciplinary team typically include the medical team, nurses, care support staff, and allied health. Allied health (AH) refers to the group of professionals from a variety of disciplines who provide direct clinical care to patients, including assessment, specialist rehabilitation, and development of individualised strategies for managing BoC when required (Australian Government Department of Health and Aged Care, 2022).

Previous research has explored the mental health and wellbeing of health professionals generally (Sharma et al., 2016; Li et al., 2021; Chidi et al., 2024; Rushton et al., 2015), and more specifically in those working with patients living with DDBoC (Costello et al., 2019; Moonga & Likupe, 2016; Pinkert et al., 2018; Turner et al., 2017). These studies suggest that working with DDBoC can have negative impacts on professional wellbeing (Gwernan-Jones et al., 2020), associated with the increased workload, stress and demands when working with this patient group (Bailey et al., 2013). Provision of dementia-specific care has also been associated with burnout in health professionals working the residential care setting (Costello et al., 2019). Burnout is characterised by an insidious erosion of capacity to perform normal duties, leading to negative effects on self, including depersonalisation, apathy and physical exhaustion (World Health Organization, 2019, World Health Organization, 2022). In healthcare work, resilience, or the ability to ‘bounce back’ after a stressful event, improves one’s capacity to manage work related stress, increase emotional wellbeing and work satisfaction (Chidi et al., 2024; Rushton et al., 2015). Studies exploring stress, burnout, resilience and wellbeing have primarily focused on nursing and medical staff, with less attention given to the impact on AH specifically.

To understand the phenomenon further, this study aimed to examine the impact of working with patients who are living with DDBoC on the wellbeing of allied health staff working in inpatient rehabilitation. The secondary aim of this study was to understand the perceived impact on AH communication and professional teamwork.

## Methods

### Study Design

This study used a mixed-methods concurrent triangulation design (Guba & Lincoln, 1981) underpinned by pragmatism, with a real-world practice orientation and a combination of inductive and deductive approaches (Guba & Lincoln, 1981). Three methods of data collection were utilised: (i) an audit of hospital patient and incident data to quantify the occurrence of workplace incidents involving allied health and patients with DDBoC from nine inpatient rehabilitation wards; (ii) a cross-sectional, online survey to capture AH staff experiences working with DDBoC; and (iii) single, semi-structured interviews were conducted to deeply understand AH staff experience.

### Setting and Participants

This study was undertaken at two sites within a major health network in Melbourne, Australia. One site was a post-acute inpatient hospital accepting rehabilitation and geriatric evaluation and management patients, and the other was an acute hospital, with two dedicated rehabilitation wards. Approximately 160 AH employees support inpatient rehabilitation services across the two sites. AH professionals are often supported by allied health assistants (AHA), important members of the multidisciplinary team, through delegated clinical and nonclinical tasks (Huglin et al., 2021). All AH and AHA staff who met the inclusion criteria were invited to participate in the survey. Eligibility criteria were: (1) currently employed at Monash Health, (2) currently working on an inpatient rehabilitation ward. There were no additional exclusion criteria beyond this. AH of all “Grades”, representing varying levels of professional experience, were included. Grade 1 staff usually equate to early-stage career clinicians, with higher Grades (2, 3, 4, etc.) reflecting higher levels of experience and responsibility in mid-late stage career clinicians, often referred to as “seniors”.

A convenience sample of AH employees who had opted in via the survey was used for primary recruitment for the interviews. Following this, the research team reviewed the sample characteristics and purposively recruited the remaining participants to obtain a maximal variation sample. Ethical approval was obtained and all participants provided written informed consent.

### Indigenous Engagement

This study was undertaken on the unceded lands of the Wurundjeri/Woi-Wurung and Boonwurrung/Bunurong people of the Kulin Nation, and the authors pay their respects to Elders past and present. Recruitment was open to all staff, and the authors did not ask participants to disclose personal demographics, including indigenous status.

### Data Collection

#### Audit

The number of patients admitted to inpatient rehabilitation wards with a diagnosis of dementia, delirium or behaviours of concern between January 2021 and December 2022 was extracted from coded medical record data. A delirium diagnosis was coded through healthcare practitioner’s admission documentation, or in the case of dementia, identified through a patient’s admission problem list, or past medical history. The data was validated by two researchers for accuracy by cross referencing medical record diagnosis data with audit data.

Incident data was extracted from the health network’s risk management system for the same time period. ‘Incident’ was defined as an occasion of occupational violence or aggression (OVA; e.g., physical or verbal aggression, discrimination or intimidation). Data were reviewed to determine the professional role of the reporter, the type of incident, and time off required due to the incident.

#### Survey

Emails were sent by departmental managers, research advisors, and senior clinicians to all AH employees working on inpatient rehabilitation wards inviting them to participate in the online survey hosted on Microsoft Forms. Responses were collected between April and June 2023. The survey was not piloted prior to responses being collected.

The survey consisted of 31 questions covering: (i) participant’s employment demographics; (ii) clinical exposure to the patient population; and (iii) support accessed (see Supplemental Materials 1). The survey included questions with dichotomous yes/no and multiple-choice response options. Three self-reported measures of stress, resilience and burnout were embedded within the survey. The *Perceived Stress Scale* (PSS) (Cohen & Williamson, 1988) and the *Brief Resilience Scale* (BRS) (Smith et al., 2008) are scales scoring self-perceived stress and recovery after a challenge, with higher scores representing greater perceived stress or resilience levels respectively. The *Abbreviated Copenhagen Burnout Inventory* (ACBI) (Barton et al., 2022) explores internal (related to personal and work-related feelings of burnout) and external (related to directly working with patients) factors of burnout separately, with thresholds determining the degree of burnout attributable to each factor. The PPS, BRS and ACBI have demonstrated validity and reliability in healthcare (Lee, 2012; Montgomery et al., 2021), blue and white collar (Soer et al., 2019) workplaces.

#### Interviews

Between April and June 2023, semi-structured interviews were conducted face-to-face at the participant’s workplace. Each participant was interviewed by one of the four research team members, all of whom have allied health clinical backgrounds, using an interview guide (see Supplemental Materials 2). The interviews were audio-recorded and transcribed verbatim. Participants selected their own pseudonym for the purpose of reporting de-identified results. The *Good Reporting of a Mixed Methods Study* (GRAMMS) checklist was used to inform the reporting of this study (O’Cathain, Murphy & Nicholl, 2008).

### Data Analysis

Data from audits, interviews and surveys were initially analysed separately, with the research team reflecting on the findings, before integrating them in a process of “*comparing, contrasting, building on*” (Creswell & Tashakkori, 2007, p. 108). Audit and survey results were integrated within the reporting of the interview themes where they offered support, tension, contrast or context to the theme. Descriptive statistics were used to analyse audit and survey data, reported as frequency and percentages. Due to the apparent differences in stress reported during the interviews, a Mann-Whitney U test and Fishers exact test were used to compare the responses on the PSS, BRS and CBI between Grade 1 and more senior staff (Grade 2 and 3) post hoc. Statistical analyses were performed using Stata (Version 18; Stata Corporation, College Station, Texas, USA). Interview data were analysed in Microsoft Excel, employing a latent content analysis process (Kleinheksel et al., 2020).

### Strategies to Enhance Rigour

The research team included AH clinician researchers employed in the same organisation as the participants. The primary and secondary researchers initially coded each interview separately, then came together to align on code and category understanding. Theme generation involved all researchers. The research team included a researcher specialising in quantitative analysis, and a researcher specialising in qualitative analysis, and both were given regular opportunities to consult, review, conduct, challenge and revise the mixed-methods design and analysis to ensure rigor. A reflective journal was kept by the primary researcher outlining methodological and analytical considerations and decisions. The study design employed triangulation with multiple data sources to “*strengthen the reliability of claims of each individual approach*” (Guba & Lincoln, 1981, p. 121).

## Results

Fifty participants, (n = 47/50; 94% female), responded to the survey, with an approximate response rate of 32% (n = 50/160). Participant demographics are represented in Table 1. Fifty-two percent (n = 26/50) of survey participants had worked in inpatient rehabilitation for greater than one year, with 28% (n = 14/50) participants being early-career clinicians (Grade 1), and 72% (n = 36/50) being mid-late career clinicians (Grade 2 or above).

**Table 1.** Participant Characteristics

	Survey participants (n = 50)	Interview participants (n = 12)
Grade		
1	14 (28%)	3 (25%)
2	24 (48%)	4 (33%)
3	9 (18%)	5 (42%)
4	3 (6%)	0 (0%)
Work site		
Casey	11 (22%)	7 (58%)
Kingston	38 (76%)	5 (42%)
Prefer not to answer	1 (2%)	0 (0%)
Gender		
Female	47 (94%)	10 (83%)
Male	3 (6%)	2 (17%)
Non binary	0 (0%)	0 (0%)
Median age (IQR)		
Years	29 (26 - 34)	N/A
Experience		
Months in subacute		N/A
3 months	7 (14%)	
6 months	11 (22%)	
12 months	6 (12%)	
>12 months	26 (52%)	
Median years in profession (IQR)	N/A	5.5 (2.5 – 8)

IQR = Interquartile range.

Semi-structured interviews were conducted with twelve AH employees, recruited by a mix of convenience and purposive sampling (to diversify participant disciplines, experience levels and gender). Their professional roles were: Occupational Therapist (OT; n = 4/12), Physiotherapist (PT, n = 3/12), Social Worker (SW; n = 2/12), AHA (n = 2/12), and Speech Pathologist (SP; n = 1/12). See Table 1 for further demographic details.

Analysis of the interview transcripts yielded four key descriptive themes: ‘*What we experience’*, ‘*How we are impacted’, ‘How we manage’* and ‘*How we work together’* (see Figure 1).

**Figure 1.**
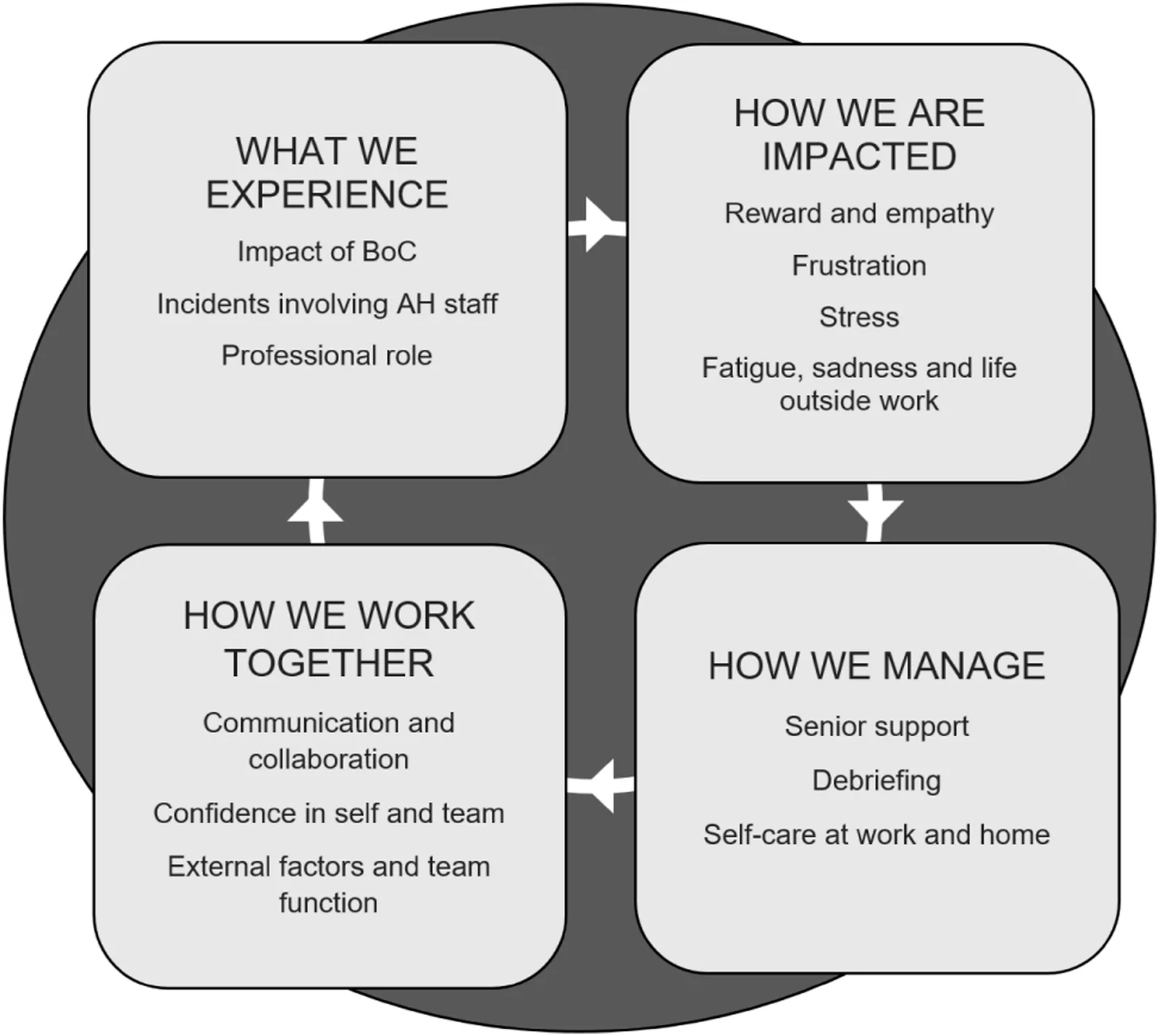
Visual representation of themes

### What We Experience: “Unfortunately Someone Has to be There to Experience That”

Forty-four percent (n = 22/50) of survey participants reported providing clinical care to five or more patients with DDBoC within the previous month (see Table 2). Similarly, interview participants reported frequent engagement with DDBoC.

**Table 2.** Exposure to Patient Population and Support

	Survey participants (n = 50)
Patients seen with diagnosis of dementia, delirium or BOC per week?	
None	2 (4%)
1-2	10 (20%)
3-4	16 (32%)
5-6	8 (16%)
7-8	6 (12%)
9-10	2 (4%)
>10	6 (12%)
Patients encountered who have exhibited BOC in the past 4 weeks?	
None	2 (4%)
1-2	14 (28%)
3-4	11 (22%)
5-6	6 (12%)
7-8	7 (14%)
9-10	3 (6%)
>10	7 (14%)
How many times diverted from task in past 4 weeks?	
None	11 (22%)
1-5	26 (52%)
6-10	10 (20%)
11-15	2 (4%)
16-20	1 (2%)
Asked for informal support in past 4 weeks?	
None	15 (30%)
Once	10 (20%)
Twice	10 (20%)
Three times	6 (12%)
Four times or more	9 (18%)
Asked for formal support in past 4 weeks?	
None	35 (70%)
Once	9 (18%)
Twice	6 (12%)
Three times	0 (0%)
Four times or more	0 (0%)

BoC = Behaviours of concern. Informal support includes support from a peer, colleague or supervisor. Formal support includes accessing an employee assistance program or structured support through a manager.

There was emphasis placed on the BoC experienced, and the impact these had on clinician time. Descriptors such as “*challenging behaviours*” (Leslie, PT), “*wandering*” (Amelia, OT), “*aggressive… very agitated*” (Meg, OT), and “*escalated*” (Ashleigh, OT) described the BoCs experienced frequently. Seventy-eight percent (n = 39/50) of survey participants reported being diverted from a work task in order to engage with a patient with BoC within the past month (see Table 2). Figure 2 describes the most commonly experienced behaviours. Seventy-five percent (n = 9/12) of interview participants shared a personal story of exposure to occupational violence and aggression whilst working with DDBoC. This included having “*had things thrown at me, been chased, yelled at”* (Sasha, SW) through to having “*had a patient that’s tried to hit me when I’ve gone to help them*” (Daisy, AHA). In contrast to the high frequency of exposure described by interview participants, audit data identified only 39 AH-reported incidents associated with patients with a dementia or delirium diagnosis over the preceding two-year period. The incidents reported were predominantly physical in nature (n = 13/39; 33%). Conversely, survey data found verbal aggression to be more frequently experienced by participants (n = 31/50), inviting speculation about the seemingly incongruent representation of incidents being formally reported. Table 1 and Figure 2 provide further details on reported incidents and experiences identified in the survey. With a potential reflection on this dissonance, Lenore (SP) suggested that even though behaviours from a patient with DDBoC are “*not coming from a place of harm… there’s this strong, hard, ‘We don’t tolerate that here’. We do tolerate that here and you watch your colleagues tolerate that.”*

**Figure 2.**
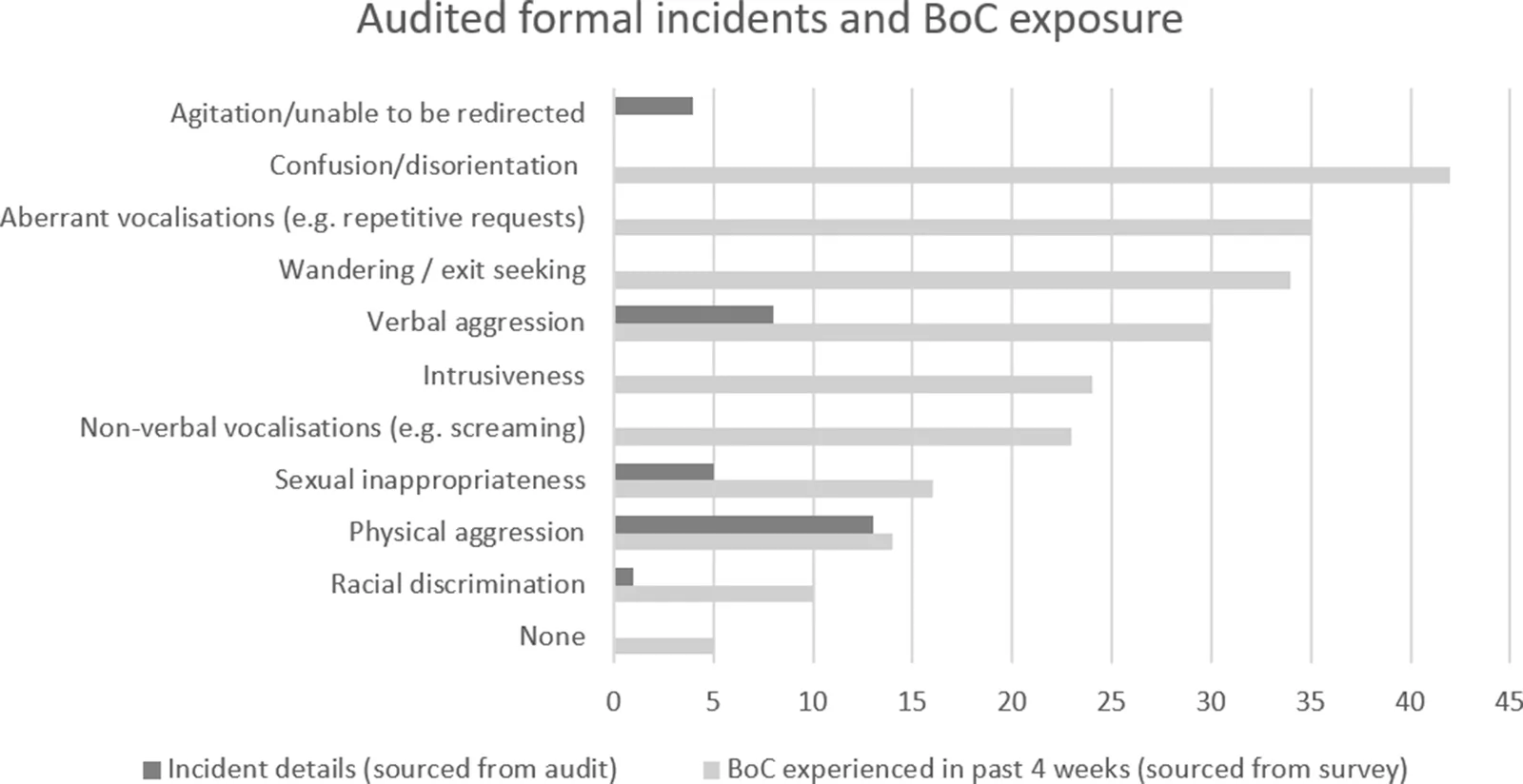
Audited formal incidents and BoC exposure data from survey. BoC = Behaviour of concern. Formal incident data sourced from audit over 2 year period. BoC exposure sourced from survey

Professional role impacted the frequency with which AH engaged with patients living with DDBoC. Some participants described flexibility in their roles depending on the patient’s needs and presentation.

“[I] wouldn’t necessarily become involved until a delirium has, I guess, resolved really… but they usually then become complex discharge planning [wise] and then that's where we are quite heavily involved” (Sasha, SW)

“Our role might be a bit more consultative, versus if they’ve got a…language impairment and that’s really correlated to their behaviours of concern, we might be a lot more involved” (Lenore, SP)

Others had more frequent contact or intimate proximity with patients as a result of their role.

“Well, I potentially don’t want to be alone with them in a shower.” (Amelia, OT)

### How We Are Impacted: “It’s Challenging, But it’s Good”

Interview participants discussed the emotional, psychological, and behavioural impact of working with this patient group. Positive experiences, including making a difference, enabling progress, and providing patient-centred care, sat alongside challenging experiences, like feeling unsafe at work, feeling stress and fatigue, and ethical tension.

Participants felt reward in “*being able to support them*” (Amelia, OT), and “*work[ing] together as a team*” (Lenore, SP). These experiences increased capacity for empathy and patient-centred care, with Leslie (PT) reflecting, “*I feel like I’m willing to take more time… just being ‘patient with the patient*’”. Ronald (OT) described the professional reward experienced when successful strategies make a difference to a patient.

“We had this patient transferred to us who'd had seven [aggressive incidents] in a week and was trying to kick down the door on another ward … But, by the end of his admission… he got to the point where the whole team was just so aware of him and knew what to do… it was amazing. So that is where the reward is because this person is genuinely having a good experience.” (Ronald, OT)

Participants described pride in their work, often smiling or laughing when sharing meaningful or funny stories, highlighting how emotion played a role in the experience of feeling work-related satisfaction.

Feelings of frustration punctuated interviewee reflections. While “*most health professionals… can understand that these behaviours of concern*...*aren’t intentionally done to hurt you or to have a negative impact on you*” (Lenore, SP), frustration came from when participants experienced a sense of unproductivity. For example, *“…when you have… a treatment goal for the session, which is for the patient’s wellbeing, of course, but you’re not really making gains with that*” (Betsy, PT). More junior therapists reflected on the complexity of this patient population affecting their “*ability to carry a caseload*” (Leslie, PT) as they needed to “*put more time into these patients*” (Meg, OT).

Frustration also arose from factors outside the clinician’s control.

“Working in a system that you can’t change necessarily, or you’re working with a family member that just you can’t get through to” (Charlie, SW)

These frustrations contributed to stress in managing workloads in this area. 30% (n = 15/50) of survey participants were experiencing abnormal levels of stress as measured by the *PSS.* Despite not being the majority, during interviews, participants delved deeper into the relationship between managing patients living with DDBoC and stress. Leslie (PT) described the challenge of “*trying to manage these behaviours … and balance therapy time*”, whilst Lenore (SP) felt that frequency of exposure to working with a patient who was *“…clinically complex multiple days in a row contribute to stress*.” Charlie (SW) shared feelings of “*fear for your personal safety, fear about doing the wrong thing*” in the workplace, and Daisy (AHA) described *“…that little minute before you walk in the patient’s door where you’re like, ‘Are you going to be okay in this situation?*’”. By contrast, Mitch (PT) reported that “*working with people with dementia and delirium hasn’t had a negative impact on my mental health*”, reflecting on his self-described “*privilege*” of being male in a context where “*ingrained sexist and generational beliefs in healthcare and the population we’re working with particularly that do maybe make my job easier in a lot of ways… [and] can bring me confidence and security.”*

There was no significant difference in stress levels between early-stage and mid-late stage career clinicians in the survey (see Table 3), however interview responses suggested that stress may manifest differently for each. Mid-late career staff reflected on the stressful aspects of their expert status.

“… the junior staff lean on me for doubles and – so my caseload with this [complex] population is more saturated” (Betsy, PT)

“You’ve got another team member coming to you saying ‘what do I do?’. And then making sure that that anxiety doesn’t transfer to you” (Charlie, SW)

“What I love about it is being able to support some of those really challenging patients and coach and spend time with junior staff… But with bed pressures and leave and things, you’ve got to just be flexible.” (Ashleigh, OT)

**Table 3.** Outcome Measure Results

	Total survey participants (n = 50)	Grade 1 participants (n = 14)	Grade 2 and above participants (n = 36)	*p* value^a^
Brief resilience scale				
Low resilience	13 (26%)	4 (29%)	9 (25%)	
Normal resilience	31 (62%)	8 (57%)	23 (64%)	*p* = 1.000^a^
High resilience	6 (12%)	2 (14%)	4 (11%)	
Perceived stress scale				
Low stress	12 (24%)	4 (29%)	8 (22%)	
Normal stress	35 (70%)	9 (64%)	26 (72%)	*p =* 0.747^a^
High stress	3 (6%)	1 (7%)	2 (6%)	
Abbreviated Copenhagen burnout inventory				
Internal factor				
Lower burnout	38 (76%)	12 (86%)	26 (72%)	*p* = 0.468^b^
Higher burnout	12 (24%)	2 (14%)	10 (28%)	
External factor				
Lower burnout	36 (72%)	10 (71%)	26 (72%)	*p* = 1.000^b^
Higher burnout	14 (28%)	4 (29%)	10 (28%)	

Based on ^a^Mann-Whitney U test and ^b^Fishers exact test; Significance was recorded as *p* < 0.05.

External factors such as “*overall workload*” (Betsy, PT), expectations, safety culture, staffing levels, organisational and systemic norms were linked to the experience of stress in the workplace.

“If I’ve had 20 meetings, and I’ve got documentation to do, and someone needs a report written on someone, then there’s going to be a lot more just like ‘I don’t have time for you to be doing this’”. (Ashleigh, OT)

Needing to balance gold standard, patient-centred care with the resources available was a commonly identified cause of stress. Instances where a lack of time lead to shortcuts being taken introduced uneasiness in AH staff.

“Maybe we’re doing what’s easier for the staff member involved most often versus what’s actually in the best interests of the person who’s exhibiting those behaviours.” (Ashleigh, OT)

Navigating “*grey areas*” (Charlie, SW), such as dignity of risk, or issues of consent were challenging ethical dilemmas to navigate. Participants acknowledged that advocating for their patients was daunting at times.

“Yes, they have dementia, yes, they have a cognitive impairment, but in the eyes of the law we have to be least restrictive. And that looks like sitting with, sometimes, a significant amount of risk… Which can be a little bit overwhelming, especially when they are really complex.” (Sasha, SW)

Participants described the need “*to be on all the time*” (Betsy, PT), or to “*juggle and fight in my head before seeing a patient like that*” (Leslie, PT) resulting in emotional or mental fatigue. For Ronald (OT), it manifested as physical fatigue and feeling “*absolutely just depleted. 110% just depleted*.”

Participants described feelings of sadness and defeat resulting in fatigue when working in this area.

“So you feel sad for them, you just care. So, I guess that can be tiring” (Chappy, AHA)

Professional roles and identity impacted on sadness experienced by AH staff. Sasha (SW) explained how emotionally charged the process of assisting a patient living with dementia to plan for supported accommodation can be, whilst Amelia (OT) expressed her grief at witnessing loss of patient independence:

“I think you can't help but feel responsible when people respond badly… And when they respond badly, feeling guilty and sad and I guess a bit of that depression.”

“Patients who– had really full lives, and been really independent, and then-they’re just not there anymore.”

Some participants felt the impact outside of working hours. Leslie (PT) described having “*found myself thinking about these patients more outside of work time and feeling like I’m not as motivated to come into work.*” Examples were shared of the impact this had on personal relationships, with Ronald (OT) describing the impact of workplace experiences “*mean that I don’t connect as much with my friends and family because I’m just knackered.”*

These examples may illustrate the lived experience of survey respondents who scored low in the BRS (n = 13/50; 26%), suggestive of low levels of resilience; or the number of participants who scored higher in the internal and external burnout factors of the ACBI (n = 12/50; 24%, n = 14/50; 28% respectively.) Conversely, there were participants who were not affected by their work outside of business hours and had strategies to ensure resilience was maintained.

### How We Manage: “Are You Okay?”

Access to support from senior staff for decision making, supervision, reflection, role modelling and feedback was a “*go-to*” (Meg, OT) strategy when working with patients with DDBoC. Adequate access to experienced senior clinicians when planning clinical management and for supporting career development was valued by participants.

“I think having more access to social work seniors… has helped as well because they've obviously experienced similar things so that they understand” (Sasha, SW)

“I had a really great supervisor when I first graduated who – she was quite resilient, and she was very empathetic but also had a strength to her I think, and I really looked up to her for that.” (Ashleigh, OT)

Seventy percent (n = 35/50) of survey participants reported seeking informal support for managing patients living with DDBoC, via their senior, peers or colleagues, while 30% (n = 15/50) had engaged with formal support through their manager or an employee assistance program. Six participants (12%) had sought this level of support twice in the past month (see Table 2).

Formal and informal debriefing was identified as a “*key*” (Charlie, SW) strategy for staff working with DDBoC. The nature of debriefing varied from structured post-session feedback, “*to reflect on how that session went, and what occurred...what went well and what didn’t go so [well], and how I feel about it*” (Betsy, PT), through to informal processes where “*you go back to your colleagues and you’re like, ‘Whew, that was hard work. Oh my gosh, what are we going to do about that?’”* (Chappy, AHA). Regardless of format or purpose, debriefing remained a pivotal strategy which participants valued highly.

When asked how they manage the impact working with DDBoC has on wellbeing, interview participants described their strategies for self-care at work and at home. At work, (Amelia, OT) put mental boundaries in place between her and her patients in order to continue her work “b*ecause there’s 20 other people on this ward, and they all need to see me.”* Similarly, Mitch (PT) recognised he “*can’t put energy into this if it is not something I can control*”. Participants shared how they “*just take a moment*” (Meg, OT) to help manage energy levels throughout the workday. Outside of business hours, participants shared their self-care strategies including meditation, walking, deep breathing, healthy relationships, and good exercise, sleep and hygiene patterns. These examples of self-management strategies may help to understand the experience of the 74% (n = 37/50) of survey respondents who scored within the normal and high ranges in the BRS, representing stronger levels of resilience.

### How We Work Together: “It’s Not My Decision. It’s Our Decision”

Communication was identified as central to team function, and participants identified that working with people with DDBoC often enhanced communication.

“Honestly, it probably promotes communication a little bit, because we have to do checking in with each other around this has happened, that’s happened, how do we want to respond to this?” (Amelia, OT)

“It probably brings us closer, weirdly enough.” (Daisy, AHA)

Participants agreed that a collaborative multidisciplinary team enhanced their ability to perform their roles cohesively. Working in a team also led to an enhanced feeling of safety, knowing that there were other people to “*keep an eye out and jump in if they see you struggling*” (Amelia, OT). There was a sense of “*camaraderie*” (Ronald, OT), with different members of the team striving towards the same goals and facing the same challenges.

Conversely, experiences of a lack of communication and collaboration was considered to negatively impact team culture and potentially increase risk to patients.

“[Poor communication] add[s] to the distress of the situation.” (Mitch, PT).

AH participants expressed their confidence in “*knowing and feeling like I have the capacity and the skills to support [people with DDBoC]”* (Ashleigh, OT), through citation of evidence-based management strategies such as use of the “*Sunflower tool*” (Meg, OT) or patient empowerment: *“…having goals within sessions, ensuring it is their idea that they want to be there to do things.*” (Betsy, PT). There was a perceived reliance on experienced members of the multidisciplinary team, often AH staff, to lead and manage complex patient care, and to support both junior staff and the broader team.

“Wards rely on people who are more experienced in those situations to take on those patients” (Lenore, SP)

Participants expressed confidence in the contributions of their fellow AH in the management of DDBoC. However, when prompted to reflect on their experience with non-AH disciplines, their responses became more hesitant. Reasons given included a perceived low level of specialised “*knowledge and education*” (Mitch, PT), and a perceived lack of role delineation in which *“...it’s tricky in terms of boundaries in roles”* (Charlie, SW). There was empathy for this work being “*quite taxing”* (Betsy, PT) on nursing staff, where they “*...have no escape*” (Lenore, SP) due to the nature of shift work, night duty and supporting care tasks.

The ability for teams to provide optimal care was perceived by participants to be impeded by factors outside their control. Ashleigh (OT) reflected on the “*staffing resource factor*” as a barrier, questioning whether additional support for diversional strategies could “*make life easier for the patient and staff.*” Budget constraints were often identified as a challenge for the implementation of recommended strategies from “*a funding perspective*” (Leslie, PT).

## Discussion

This study explored the impact working with patients living with dementia, delirium or behaviours of concern has on the wellbeing of allied health staff in inpatient rehabilitation. Survey and interview responses revealed the impact was complex, with a range of positive and negative feelings affecting levels of stress, burnout and resilience. There was also an effect on team function. This study adds a novel contribution by reporting on the unique experience of AH staff, extending on previous research involving mixed hospital staff cohorts (Clissett et al., 2014; Duah-Owusu White & Kelly, 2023; Gwernan-Jones et al., 2020), nurses (Digby et al., 2017; Moonga & Likupe, 2016; Pinkert et al., 2018), and healthcare assistants (Bailey et al., 2013).

Participants described their work in this area to be hard, but rewarding in instances where patient-centred care was able to be delivered. In an ethnographic study by Bailey and others (2013), healthcare assistants similarly found working in the dementia area to be emotionally burdensome yet rewarding when able to engage in a physically involved, cognitively vigilant and emotionally connected way with patients. In the present study, AH participants described working in this area as challenging, especially with respect to BoC, and the impact this had on stress, fatigue and sadness. A systematic review by Moonga and Likupe (2016) reported on the links between BoC in patients admitted with dementia and increased workload, leading to increased stress and burnout in nurses. It appears that this impact is consistent between nursing, healthcare assistants and AH staff.

Variability in the impact of stress was evident in this study. Survey results found 70% of participants had normal stress scores, and 4% of participants reflected a state of high stress. Despite there being no significant differences in stress outcomes between early and mid-late-career staff, interview responses suggested that they experience stress differently. Seniors felt they often managed more complex patients, with expectations to support their junior colleagues and deliver departmental priorities within operational constraints, contributing to higher levels of stress working in this area. This is consistent with the findings of Turato et al. (2022), who identified that navigating complex hospital systems and a lack of leadership training may contribute to AH teams being at an increased risk of stress, burnout and poor morale. Given the potential for stress to manifest more insidiously within senior staff, monitoring and investing in this cohort could be considered to mitigate adverse outcomes.

Professional role appeared to have an impact on the experience of stress. Where AH roles were more consultative, an opportunity to detach from the case was evident (for example, in instances where SP or AHA roles were less involved with the patient). For others, their intimacy with the patient’s situation brought on feelings of stress. SW participants shared their experiences of managing decision making capacity in patients where cognition is questioned, leading to challenges in patient advocacy and professional ethical strain. This finding echoes the results of a qualitative interpretive meta-analysis by (Chen & Highfill, 2023), who found that SWs can experience emotional stress as a result of tension between professional and personal values in dementia care.

The experience of burnout, stress and eroded resilience appeared to be entwined with the ability of AH to provide patient-centred care. Where tension between perceived best care and actual care existed, feelings of emotional and physical fatigue, burnout and insidious effects on life outside work were described. This echoes the findings of a systematic review by Gwernan-Jones and colleagues (2020) which found that where person-centred care could not be delivered, staff may experience moral distress. Extra time and clinical reasoning burden in this cohort, especially for junior staff, feelings of being unsafe, staffing limitations, financial constraints, and task-focused care being prioritised were all cited by participants as hurdles to patient-centred care. Similar operational constraints have been described by Duah-Owusu White and Kelly (2023) and others (Bailey et al., 2013; Digby et al., 2017; Gwernan-Jones et al., 2020; Moonga & Likupe, 2016; Pinkert et al., 2018; Turner et al., 2017). Different barriers to patient-centred care have been observed in other studies. Clissett et al. (2014), described how health professionals perceived patients with DDBoC to be a disruption on the ward, eliciting a range of helpful or harmful responses to their increased needs. Bailey et al. (2013) observed a depersonalisation of the patient with dementia by health professionals where internal emotional capacity was reduced.

Whilst high exposure to BoC was reported in both survey and interview responses, there appeared to be an incongruence in the rate of formal incident reporting. Verbal aggression (behaviour warranting reporting) was the most frequently experienced by survey participants, yet predominantly physical incidents were reported. This is a similar finding to Arnetz et al. (2015), who found a mismatch between informal and formal incident reporting, with physical and lost time injuries being more frequently reported due to mandatory hospital policy. Previous evidence has established factors which may influence underreporting: a lack of local incident reporting culture, time constraints, shorter time employed in role, minimisation of perception of harm, or a belief that the behaviour is a function of the disease process affecting the patient and not intentional (Arnetz et al., 2015; Van Fossen & Chang, 2022). This may explain statements such as, “*it’s not coming from a place of harm*” (Lenore, SP) expressed by our participants, suggesting that AH may under-report incidents of workplace violence and aggression when working with patients living with DDBoC as they may not perceive the behaviour as deliberate or malicious. Organisers of education and preventative measures aimed at staff safety informed by accurate reporting of incidents (Arnetz et al., 2015) should be mindful of potential underreporting in this group.

Consistent with previous studies (Duah-Owusu White & Kelly, 2023; Moonga & Likupe, 2016), AH staff in this study found patients with DDBoC required additional time and resources from the whole treating team. Whilst working in a team, AH expressed confidence in their own ability to manage patients with DDBoC, as well as their fellow AH disciplines. There was reduced confidence in other members of the multidisciplinary team. Participants cited a lack of specialised training and time available to nursing colleagues as reasons for their reduced confidence, two factors found by Turner et al. (2017) to be barriers for nurses delivering person-centred care in dementia. A narrative review by Duah-Owusu White and Kelly (2023) found related themes of professional mistrust and differing opinions. The scope of this project limited the investigators in understanding this phenomenon from other disciplines’ perspectives, and further work may be required to determine how it may be overcome. In the present study, strong communication and team-based collaboration were held as key touchstones by participants for the team to experience success together, and may increase interdisciplinary confidence, inviting the possible value of interdisciplinary training to strengthen team performance (Duah-Owusu White & Kelly, 2023; Roth & Morkova, 2012).

Our participants explored strategies for maintaining wellbeing working with DDBoC. Access to adequate support from senior staff and debriefing, which served multiple functions for our participants as a form of feedback, validation and ventilation (Duah-Owusu White & Kelly, 2023; Iosim et al., 2021) were emphasised. Strategies to enhance wellbeing inside and outside of the workplace identified by our participants included healthy boundary setting; taking breaks and general self-care strategies, some of which have previously been described (Burner & Spadaro, 2022; Leiss et al., 2021). Hospital leadership and organisation stakeholders are well placed to invest in interventions which support employee wellbeing, with individual level strategies (including mindfulness, yoga and gratitude) shown to improve resilience, stress and reduce burnout in health professionals. However, organisation-level strategies may make a larger impact (Caballol-Avendaño et al., 2021; Cohen et al., 2023; McPherson et al., 2016; Perlo et al., 2017). Given the positive impact of being able to provide patient-centred care found in this study, organisational priority on the delivery of dementia friendly, patient-centred care may enhance staff wellbeing and job satisfaction (Bailey et al., 2013; Gwernan-Jones et al., 2020).

The majority of participants in this study identified as female (Survey n = 47/50; 94%; interview n = 10/12; 83%) potentially leading to gender biased findings. Higher female representation is consistent with workforce demographics and study context, rather than a result of sampling bias. Previous literature has demonstrated a disparity in female health professionals experiencing workplace violence and aggression, and gendered work may have impacted on the type, frequency and experience of behaviours encountered by our participants (Ayaz et al., 2024; Nelson et al., 2024). Younger age and low years of experience have both been identified as risk factors for violence in healthcare (Nelson et al., 2024), which may have influenced the experience of our participants (median age 29 years, 48% (n = 24) with <12 months experience in subacute).

### Limitations

Limitations exist within this study which restrict generalisability of results, including the survey response rate of 32% of eligible staff. This study took place in a single health network in Australia; future research may be strengthened through multi-site designs. Statistical analysis of outcomes between disciplines was not undertaken due to low sample sizes, but future work could leverage this insight to inform targeted training or interventions. While outcome measures embedded in the survey had established validity and reliability in similar populations, individual AH professions included in the study had not been studied. Lastly, as this study employed a concurrent triangulation design, interview questions were not based on survey responses, which may have guided more targeted exploration of themes. However, the use of three data sources reduces the dependency on a particular data type and adds strength to the results. Overly direct lines of questioning about potentially negative topics such as perceived participant resilience and burnout levels were avoided to minimise risk of participant distress.

## Conclusion

This study explored the impact working with patients living with dementia, delirium and/or behaviours of concern had on AH staff wellbeing and teamwork in a hospital setting. The results provide valuable insights into the unique lived experience of AH professionals working in this area. Engagement with patients with DDBoC is frequent; BoC are experienced regularly by AH staff, however incidents may be under-reported formally. Normal levels of stress and burnout were present in the sample majority, however a small proportion of AH staff were found to be experiencing higher levels of stress, burnout and eroded resilience. Stress may manifest differently in junior and senior AH staff, and some professional roles. AH may experience enhanced work related wellbeing where patient-centred care can be delivered as a team. Leadership stakeholders should consider the complex impact working with DDBoC has on AH staff. Adequate training, resources and supports; emphasis on cognitively safe, patient-centred care; organisational and individual-based wellbeing interventions to support AH staff wellbeing should be prioritised. Further research is required to understand how wellbeing strategies may impact both staff wellbeing and patient outcomes in this important and growing clinical area.

## Supplemental Material

**Supplemental Material -** Working With Patients Living With Dementia, Delirium and Behaviours of Concern: An Exploration into the Impact on Allied Health Staff Wellbeing Supplemental Material for Working With Patients Living With Dementia, Delirium and Behaviours of Concern: An Exploration into the Impact on Allied Health Staff Wellbeing by Taree Gibson, Melissa Roberts, Sarah C. Milne, Abby M. Foster in Dementia.

## Data Availability

Raw data are not publicly available to preserve participant privacy.
