# Supplementary material for: Working With Patients Living With Dementia, Delirium and Behaviours of Concern: An Exploration into the Impact on Allied Health Staff Wellbeing
Source: Dementia (London). 2026 Jan 12;25(8):2000–19. doi: 10.1177/14713012251408675 (PMC13683904; doi:10.1177/14713012251408675)
Supplement: Supplementary file 1 [file sj-pdf-1-dem-10.1177_14713012251408675.pdf]

# Supplementary materials

## *Supplementary material 1. Survey*

| Domain | Question | Answer/s |
|---|---|---|
| Demographics | Participant Information Statement | – I do NOT consent to participate (skip to end of survey if selected)<br>– I consent to participate |
| | Which discipline are you? | – Physiotherapist<br>– Occupational Therapist<br>– Dietitian<br>– Speech Pathologist<br>– Podiatrist<br>– Social Worker<br>– Music Therapist<br>– Exercise Physiologist<br>– Allied Health Assistant<br>– Other (please specify)<br>– Prefer not to answer |
| | What is your Grade? | – 1<br>– 2<br>– 3<br>– 4<br>– Prefer not to answer |
| | How long have you worked in subacute for? | – 3 months<br>– 6 months<br>– 12 months<br>– > 12 months |
| | Which site do you work at? | – Kingston<br>– Casey<br>– Prefer not to answer |
| | What is your year of birth? | – Display options 1900-2020<br>– Prefer not to answer |
| | What is your gender? | – Male<br>– Female<br>– Non-binary |

| | | |
|---|---|---|
| | | – I identify as: _____________ (please specify).<br>– Prefer not to answer |
| **Exposure** | How many patients would you see per week (on average) who have a diagnosis of dementia or delirium? | – None<br>– 1-2<br>– 3-4<br>– 5-6<br>– 7-8<br>– 9-10<br>– >10 |
| | Thinking about the past 4 weeks; how many patients have seen who have exhibited behaviours of concern? | – None<br>– 1-2<br>– 3-4<br>– 5-6<br>– 7-8<br>– 9-10<br>– >10 |
| | Thinking about the past 4 weeks, which of the following have you experienced when working directly with your patients who have a diagnosis of dementia or delirium? | Select all that apply:<br>– Verbal aggression<br>– Aberrant vocalisations (repetitive requests)<br>– Non-verbal vocalisations (e.g., screaming)<br>– Physical aggression<br>– Wandering / exit seeking<br>– Confusion / disorientation<br>– Intrusiveness<br>– Sexual inappropriateness<br>– Racial discrimination |
| | Thinking about the past 4 weeks, how many times have you needed to divert from the task you were performing in order to intervene in a situation with a patient who was exhibiting behaviours of concern? | – None<br>– 1-5<br>– 6-10<br>– 11-15<br>– 16-20<br>– >21 |
| | Thinking about the past 4 weeks, how many times have you needed to ask for informal support from a colleague, peer or senior when treating a patient living with dementia, delirium and/or exhibiting | – None<br>– Once |

| | | |
|---|---|---|
| | behaviours of concern? Support could be clinical or wellbeing in nature. | – Twice<br>– Three times<br>– Four times or more. |
| | Thinking about the past 4 weeks, how many times have you needed to ask for formal support from a dedicated assistance program when treating a patient living with dementia, delirium and/or exhibiting behaviours of concern? | – None<br>– Once<br>– Twice<br>– Three times<br>– Four times or more. |
| **BURNOUT**<br><br>**Abbreviated Copenhagen Burnout Inventory (CBI)** | How often do you feel tired? | – Never/almost never<br>– Seldom<br>– Sometimes<br>– Often<br>– Always |
| | How often are you physically exhausted? | – Never/almost never<br>– Seldom<br>– Sometimes<br>– Often<br>– Always |
| | Do you feel burned out because of your work? | – To a very low degree<br>– To a low degree<br>– Somewhat<br>– To a high degree<br>– To a very high degree |
| | Do you feel worn out at the end of the working day? | – Never/almost never<br>– Seldom<br>– Sometimes<br>– Often<br>– Always |
| | Do you find it is hard to work with patients? | – To a very low degree<br>– To a low degree<br>– Somewhat<br>– To a high degree<br>– To a very high degree |

| | | |
|---|---|---|
| | Do you find it frustrating to work with patients? | – To a very low degree<br>– To a low degree<br>– Somewhat<br>– To a high degree<br>– To a very high degree |
| **RESILIENCE**<br><br>**Brief Resilience Scale (BRS)** | I tend to bounce back quickly after hard times | – Strongly disagree<br>– Disagree<br>– Neutral<br>– Agree<br>– Strongly agree |
| | I have a hard time making it through stressful events | – Strongly disagree<br>– Disagree<br>– Neutral<br>– Agree<br>– Strongly agree |
| | It does not take me long to recover from a stressful event | – Strongly disagree<br>– Disagree<br>– Neutral<br>– Agree<br>– Strongly agree |
| | It is hard for me to snap back when something bad happens | – Strongly disagree<br>– Disagree<br>– Neutral<br>– Agree<br>– Strongly agree |
| | I usually come through difficult times with little trouble | – Strongly disagree<br>– Disagree<br>– Neutral<br>– Agree<br>– Strongly agree |
| | I tend to take a long time to get over set-backs in my life | – Strongly disagree<br>– Disagree<br>– Neutral<br>– Agree |

| | | |
|---|---|---|
| | | – Strongly agree |
| **STRESS**<br><br>**Perceived Stress Scale (PSS)** | In the last month, how often have you been upset because of something that happened unexpectedly? | – Never<br>– Almost never<br>– Sometimes<br>– Fairly often<br>– Very often |
| | In the last month, how often have you felt that you were unable to control the important things in your life? | – Never<br>– Almost never<br>– Sometimes<br>– Fairly often<br>– Very often |
| | In the last month, how often have you felt nervous and stressed? | – Never<br>– Almost never<br>– Sometimes<br>– Fairly often<br>– Very often |
| | In the last month, how often have you felt confident about your ability to handle your personal problems? | – Never<br>– Almost never<br>– Sometimes<br>– Fairly often<br>– Very often |
| | In the last month, how often have you felt that things were going your way? | – Never<br>– Almost never<br>– Sometimes<br>– Fairly often<br>– Very often |
| | In the last month, how often have you found that you could not cope with all the things that you had to do? | – Never<br>– Almost never<br>– Sometimes<br>– Fairly often<br>– Very often |
| | In the last month, how often have you been able to control irritations in your life? | – Never<br>– Almost never |

| | | |
|---|---|---|
| | | – Sometimes<br>– Fairly often<br>– Very often |
| | In the last month, how often have you felt that you were on top of things? | – Never<br>– Almost never<br>– Sometimes<br>– Fairly often<br>– Very often |
| | In the last month, how often have you been angered because of things that happened that were outside of your control? | – Never<br>– Almost never<br>– Sometimes<br>– Fairly often<br>– Very often |
| | In the last month, how often have you felt difficulties were piling up so high that you could not overcome them? | – Never<br>– Almost never<br>– Sometimes<br>– Fairly often<br>– Very often |
| **Conclusion and Sign on for Interview** | Thank you for completing this survey. If you are agreeable to participating in an interview with one of the research team to discuss these themes further, please click on this link, which will take you to another form where you can register your email address. Please note that the form embedded in this link is not related to this survey form, and that your responses given within this survey are and will remain anonymous. | – Link to second form where participants can register their email address for participation in the interview.<br>– Exit survey screen. |

*Supplementary material 2. Interview guide*

1. What is your Allied Health discipline?
2. What is your Grade?
3. How long have you worked in the Monash Health subacute space?
4. Can you describe the general patient population you work with?
5. Please describe the ward environment and team structure where you work. Who are the main team members that you work with in your ward?
6. Can you tell me about some of the behaviours of concern you have experienced whilst working with patients living with dementia or delirium in this work environment?
    a. How often do you experience these behaviours of concern?
    b. What is the most common behaviour of concern on the ward where you work?
7. What are some of the positives and negatives to working with patients living with dementia or delirium?
8. Do you ever find it stressful working with this patient group?
    a. Tell me more about your experience.
9. Do you ever experience feelings of anxiety, depression, burnout, fatigue, mood changes or brain fog working with this patient group?
    a. Tell me more about your experience.
10. Are there any strategies you use to manage your own mental health and wellbeing whilst working in this environment?
11. Have you ever felt you have needed to take time off work as a result of working with patients exhibiting behaviours of concern?
12. Who are the main team members that you work with in your ward?
13. How do you manage patients who are living with dementia, delirium or manifesting behaviours of concern as a team?
    a. How do you communicate as a team when managing this patient group?
    b. Does working with this patient group have any effect on your communication as a team?
14. How would you describe your overall confidence in managing patients who are living with dementia, delirium or exhibiting behaviours of concern?

*Supplementary material 3. Good Reporting of a Mixed Methods (GRAMMS) as described by O'Cathain, Murphy, Nicholl (2008)*

| **Guideline** | **Section: page** |
|---|---|
| Describe the justification for using a mixed methods approach to the research question | Methods, Study design: p.6 |
| Describe the design in terms of the purpose, priority and sequence of methods | Methods, Study design: p.6<br>Methods, Data Collection: p.7-8 |
| Describe each method in terms of sampling, data collection and analysis | Methods, Data Collection: p.7-8 |
| Describe where integration has occurred, how it has occurred and who has participated in it | Methods, Data Analysis: p.8<br>Methods, Strategies to enhance rigour: p.8 |
| Describe any limitation of one method associated with the present of the other method | Discussion, Limitations: p.18 |
| Describe any insights gained from mixing or integrating the methods | Results: p.9, paragraph 2<br>Results: p.11, paragraph 3-4<br>Results: p.13, paragraph 3<br>Results: p.13, paragraph 4<br>Results: p.14, paragraph 1,3<br>Discussion: p.16, paragraph 3,6 |
